# Does a new case-based payment system promote the construction of the ordered health delivery system? Evidence from a pilot city in China

**DOI:** 10.1186/s12939-024-02146-y

**Published:** 2024-03-14

**Authors:** Huanyu Shi, Zhichao Cheng, Zhichao Liu, Yang Zhang, Peng Zhang

**Affiliations:** 1 School of Economics and Management, Beihang University, Beijing, 100191 China; 2 The Second Affiliated Hospital of Shandong First Medical University, Tai’an 271000, China; 3 Tai’an Healthcare Security Administration, Tai’an, 271000 China; 4 China Reform Health Management and Services Group Co., Ltd, Beijing, 100028 China

**Keywords:** Case-based payment, Ordered health delivery system, Diagnostic intervention package, China, Provider behavior

## Abstract

**Background:**

The construction of the ordered health delivery system in China aims to enhance equity and optimize the efficient use of medical resources by rationally allocating patients to different levels of medical institutions based on the severity of their condition. However, superior hospitals have been overcrowded, and primary healthcare facilities have been underutilized in recent years. China has developed a new case-based payment method called “Diagnostic Intervention Package” (DIP). The government is trying to use this economic lever to encourage medical institutions to actively assume treatment tasks consistent with their functional positioning and service capabilities.

**Methods:**

This study takes Tai’an, a DIP pilot city, as a case study and uses an interrupted time series analysis to analyze the impact of DIP reform on the case severity and service scope of medical institutions at different levels.

**Results:**

The results show that after the DIP reform, the proportion of patients receiving complicated procedures (tertiary hospitals: β_3_ = 0.197, *P* < 0.001; secondary hospitals: β_3_ = 0.132, *P* = 0.020) and the case mix index (tertiary hospitals: β_3_ = 0.022, *P* < 0.001; secondary hospitals: β_3_ = 0.008, *P* < 0.001) in tertiary and secondary hospitals increased, and the proportion of primary-DIP-groups cases decreased (tertiary hospitals: β_3_ = -0.290, *P* < 0.001; secondary hospitals: β_3_ = -1.200, *P* < 0.001), aligning with the anticipated policy objectives. However, the proportion of patients receiving complicated procedures (β_3_ = 0.186, P = 0.002) and the case mix index (β_3_ = 0.002, *P* < 0.001) in primary healthcare facilities increased after the reform, while the proportion of primary-DIP-groups cases (β_3_ = -0.515, *P* = 0.005) and primary-DIP-groups coverage (β_3_ = -2.011, *P* < 0.001) decreased, which will reduce the utilization efficiency of medical resources and increase inequity.

**Conclusion:**

The DIP reform did not effectively promote the construction of the ordered health delivery system. Policymakers need to adjust economic incentives and implement restraint mechanisms to regulate the behavior of medical institutions.

**Supplementary Information:**

The online version contains supplementary material available at 10.1186/s12939-024-02146-y.

## Introduction

Under a severe medical resource environment, providing basic medical care services for as many people as possible has become a huge challenge for the Chinese government [1]. Although the health status of Chinese nationals has improved in recent years, there is still a massive gap in per capita medical resources compared with developed countries, which has resulted in many patients being unable to receive appropriate treatment [2, 3]. Meeting this challenge necessitates enhanced healthcare service efficiency and reduced inequality [4, 5].

China implements a three-level hierarchical healthcare system consisting of tertiary hospitals, secondary hospitals, and primary healthcare facilities. During the planned economy period, residents who need medical treatment should first be treated in primary healthcare facilities. Those unable to obtain adequate treatment would then be transferred to superior hospitals. Despite constraints on medical capacity at that time, this system undeniably ensured patients’ rights to non-competing medical resources [6, 7]. Since the transition from a planned economy to a market economy in the 1980s, the emphasis on social equity has gradually given way to economic development efficiency. The distribution of medical resources is increasingly irrational, disrupting the orderly flow of patient groups among medical institutions at different levels. An increasing number of patients with minor illnesses are flooding into superior hospitals, resulting in terrible wastage of medical resources that would ideally be reserved for treating severe cases [8, 9]. Consequently, medical treatment becomes challenging and expensive for patients in rural areas. [10]. Therefore, reconstructing the ordered health delivery system is crucial for ensuring health equity and improving medical service efficiency.

Since the new medical reform in 2009, the Chinese government has made great efforts to construct an ordered health delivery system. In September 2015, the General Office of the State Council issued the “Guiding Opinions on Promoting the Construction of a Hierarchical Diagnosis and Treatment System”, proposing to establish a treatment protocol of “initial diagnosis at the primary level, two-way referral, separating diagnosis and treatment of acute and chronic diseases, upper and lower linkage” by 2020 [11]. This initiative aims to encourage the implementation of specialization and collaboration among medical institutions across various levels, promoting the establishment of an efficient division of labor system [12]. Under the new health delivery system, medical institutions are expected to assume treatment tasks consistent with their functional positioning and service capabilities [13]. Patients with common, minor, and chronic diseases should receive treatment from primary healthcare facilities, while patients with serious diseases should go to superior hospitals for treatment [14, 15]. However, the absence of gatekeeper systems, combined with the inadequate quality of primary healthcare facilities, frequently results in patients with minor diseases bypassing primary healthcare facilities and directly seeking care in superior hospitals [16–21].

The crucial role of medical insurance in restructuring the ordered health delivery system has gained growing interest among researchers. Firstly, medical insurance has a regulating effect on the behavior of patients. Previous research has demonstrated that widening the gap in reimbursement rates between primary healthcare facilities and superior hospitals encourages patients to choose primary healthcare facilities for their treatment [22]. In addition, in some areas, the medical insurance reimbursement threshold for the second hospitalization fee of downwardly referred patients is exempted, promoting two-way referral [23]. Secondly, as an essential economic leverage, the medical insurance payment method influences the behavior of medical institutions [24]. For example, traditional fee-for-service incentives encourage superior hospitals to treat patients with minor illnesses as they can maximize their revenue by offering unnecessary services [25, 26]. In recent years, China has implemented various payment methods, among which a new case-based payment system called the “Diagnostic Intervention Package” (DIP) has attracted much attention.

DIP is a Chinese original payment method. The Chinese government is making significant efforts to advance DIP reforms in an attempt to influence the behavior of healthcare providers positively [27, 28]. While previous studies have examined the impact of DIP payment reform on per-admission spending, out-of-pocket spending, length of stay, and quality of healthcare in Chinese hospitals, there has been a notable absence of discussion regarding whether the case-based payment is conducive to promoting the construction of the ordered health delivery system, especially the ordered division of labor among medical institutions at different levels [29–33]. This omission stems from prior research disregarding the influence of the Medical Institution Rank Coefficient (MIRC) within the DIP system, which reflects differences in overall costs among medical institutions. A higher MIRC implies increased medical insurance revenue for the institution. The DIP system incorporates specific minor diseases into the primary DIP groups, characterized by the MIRC higher than that of primary healthcare facilities but lower than secondary and tertiary hospitals (a detailed introduction of the DIP payment system was provided in Sect. " Institutional background"). The government strives to utilize this incentive to encourage hospitals to take proactive measures to modify their service scopes, prioritizing severe cases in superior hospitals and designating primary healthcare facilities for patients with minor illnesses. It seems achievable in Chinese hospitals that, despite medical institutions are not allowed to select patients, physicians can discuss with outpatients whose needs fall beyond the service scope of the hospital, advising them to seek admission to a more suitable medical institution [34–36]. However, whether an ideal health delivery system can be actualized through DIP payment reform remains unconfirmed.

Therefore, this study takes Tai’an, a city in eastern China, as a case study and uses interrupted time series analysis (ITSA) to assess the impact of DIP reform on the severity of treatment cases and service scope of different levels of medical institutions. The aim is to evaluate whether the existing DIP system effectively promotes the construction of the ordered health delivery system. The findings of this study offer valuable insights for policymakers to craft suitable incentives following the implementation of DIP payment.

## Institutional background

Like numerous countries, China is witnessing a gradual rise in medical expenditures, leading to an escalating risk of deficits for the medical insurance fund. A significant factor contributing to this trend is the inappropriate conduct of healthcare providers [37]. The Chinese government has taken many measures to regulate the behavior of medical providers, including payment method reform for inpatient care. In 2009, the Chinese government launched a payment system reform transitioning from retrospective payment to prospective payment methods. As part of this initiative, the Chinese government introduced the Diagnosis Related Groups (DRG) payment, which has been widely adopted in many countries. The first DRG pilot program was implemented in Beijing in 2012 [38]. Although DRG-based payment systems have proven beneficial in reducing expenditure per admission, the adoption of DRG is not widespread in China [39]. The data quality requirements are high in the early stage of DRG implementation, and the formation of the DRG grouping scheme requires a lot of medical arguments, which is a great challenge for Chinese medical institutions at the present stage [40].

To regulate the behavior of medical providers more effectively, based on DRG payment, the Chinese government has developed an innovative payment method called the “Diagnostic Intervention Package” (DIP). The DIP payment system can automatically generate a disease grouping scheme through a program based on historical data and established rules, thereby reducing dependence on the level of medical development. In 2020, the National Healthcare Security Administration selected 71 cities, including Tai’an, as the first batch of DIP reform pilots [41]. Tai’an is a city in eastern China, central Shandong Province, with a permanent population of 5.472 million in 2020. The city’s per capita GDP ranked 12th among the 16 prefecture-level cities in Shandong Province at 50,576 RMB in 2020, which is lower than the provincial average (71,825 RMB) and the national average (71,828 RMB) [42]. The urban employee basic medical insurance (UEBMI) and urban resident basic medical insurance (URBMI) are the two pillars of the social insurance system in Tai’an. The two insurances cover more than 95% of the city’s population.

**Fig. 1:**
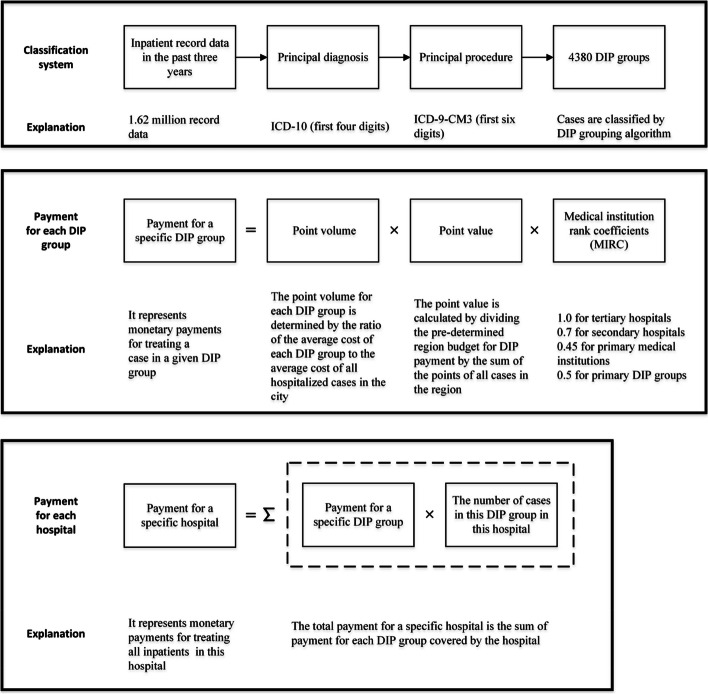
Core components of payment method by Diagnosis Intervention Packet (DIP)

In October 2021, the Tai’an Healthcare Security Administration launched the reform of the DIP payment for inpatient services, replacing the original fee-for-service payment. Figure 1 provides a schematic diagram of the Tai’an DIP payment system. In May 2021, according to the instructions of the National Healthcare Security Administration, the Tai’an Healthcare Security Administration collected inpatient record data from medical institutions in the city in the past three years. Following two rounds of matching and data cleaning, a total of 1.62 million records that satisfied the DIP grouping criteria were obtained. Inpatient cases are classified by their principal diagnosis and the procedure performed. The first four digits of the International Classification of Diseases, 10th Revision (ICD-10) are used to identify the principal diagnosis of the cases, and the first six digits of the International Classification of Diseases, 9th revision, Clinical Modification (ICD-9-CM3) are used to identify the principal procedure of the cases. The sample from the preceding three years was pre-grouped, forming 4380 DIP groups. DIP covers almost all inpatient cases treated in both public and private hospitals in Tai’an, except for mental diseases.

Each DIP group is assigned a specific point volume. The point volume is a weight given based on the resource consumption of each group, which reflects the severity of the disease and the complexity and difficulty of the treatment. The point volume for each DIP group is determined by the ratio of the average cost of each group (weighted at 70%, 20%, and 10% for 2020, 2019, and 2018, respectively) to the average cost of all hospitalized cases in the city from 2018 to 2020:

1 $$P = \frac{{C_{2020} \times 70\% + C_{2019} \times 20\% + C_{2018} \times 10\% }}{{C_{3} }} \times 1000$$

where $P$ is the point volume of a specific DIP group; $C_{2020}$, $C_{2019}$, $C_{2018}$ are the average costs of a specific DIP group in 2020, 2019, and 2018, respectively; $C_{3}$ is the average cost of all hospitalized cases in the city from 2018 to 2020.

The actual monetary payments for a specific DIP group are determined ex-post based on the point value. The point value is calculated by dividing the pre-determined region budget for DIP payment by the sum of the points of all cases in the region. Therefore, the actual reimbursement for a specific medical institution is affected by the point volumes of other medical institutions in the market, establishing a competitive relationship among various medical institutions.

Service costs for treating the same disease differ among various medical institutions in China due to considerable variations in the consumption of medical resources across different levels of these institutions [11]. The MIRC can be regarded as the proportional relationship between the comprehensive resource consumption of different medical institutions for the treatment of insured inpatients. The scientific and reasonable determination of the MIRC is related to the fairness of payment for providers. Tai’an Healthcare Security Administration sets the MIRC by comprehensively considering the resource consumption of medical institutions, as well as the functional positioning, medical treatment capabilities, and specialty characteristics of these institutions. The determination process of MIRC reflects support and preference for primary healthcare facilities, aiming to encourage them to improve their service capabilities and quality of care. Initial proposals recommended a MIRC of 1 for tertiary hospitals, 0.67 for secondary hospitals, and 0.4 for primary healthcare facilities. However, this plan was not put into action, as managers of primary healthcare facilities foresaw challenges in competing with superior hospitals and anticipated financial losses. Following two rounds of seeking opinions, with particular emphasis on input from primary healthcare facilities, the MIRC for tertiary hospitals, secondary hospitals, and primary healthcare facilities were ultimately set at 1, 0.7, and 0.45, respectively. In addition, 150 primary DIP groups have been established, comprising common, frequently-occurring, and chronic diseases (Table S1 in Additional file 1). Given their relatively stable medical expenses and the frequent need for conservative treatment or uncomplicated procedures, cases within the primary DIP groups are well-suited for treatment in primary healthcare facilities. There is no difference in the MIRC of medical institutions at different levels for treating cases within primary DIP groups. The MIRC of primary DIP groups is set to 0.5, higher than that of primary healthcare facilities and lower than that of secondary and tertiary hospitals. Therefore, primary care facilities can obtain more profits by treating patients in the primary DIP group than other groups. Conversely, secondary and tertiary hospitals treating patients in the DIP group may incur financial losses. The government hopes that by granting undifferentiated MIRC to primary DIP groups across various medical institutions, superior hospitals will actively direct patients with minor illnesses to local primary healthcare facilities for treatment, and primary healthcare facilities will have a stronger willingness to treat patients within primary DIP groups. In other words, after the DIP payment reform, medical institutions can maximize their profits by providing services consistent with their service capabilities and functional positioning.

As shown in Fig. 1, the product of the point volume, point value, and MIRC is the payment for a specific DIP group. If cases in a specific hospital cover t DIP groups, the total payment for the hospital is:

2 $$P{\text{ay}}ment \, for \, hospital{ = }\sum\limits_{i = 1}^{t} {Payment \, for} \, DIP \, group_{i} \times Number \, of \, cases \, in \, group_{i}$$

## Methods

### Data

The data for this study comes from the information management system of the Tai’an Healthcare Security Administration. We used a code random sampling strategy to select 50% of the contracted medical institutions in Tai’an, including 6 tertiary hospitals, 21 secondary hospitals, and 64 primary healthcare facilities. We collected de-identified patient discharge data from April 2020 to March 2023 for each selected medical institution, including 18 months before the intervention (April 2020 to September 2021) and 18 months after the intervention (October 2021 to March 2023). During this period, Tai’an implemented no other major healthcare policy reforms. Discharge data includes patient characteristics (e.g., age, gender, insurance type), hospital name, hospital level, diagnosis, procedure, etc.

In June 2023, we conducted in-depth interviews with three heads of the Tai’an Healthcare Security Administration to gain insights into the implementation of the policy. We additionally chose managers from two tertiary hospitals, two secondary hospitals, and two primary healthcare facilities in Tai’an for interviews to observe the attitudes and response measures of medical institutions at different levels regarding DIP payment. At the same time, we also conducted interviews with the heads of the Healthcare Security Administration of Guangzhou, Zhuhai, Xuzhou, and Jinhua because these cities implemented payment method reforms before 2021 and accumulated operational experience [29, 33, 41].

### Outcome variables

Wei et al. developed a bi-objective model incorporating medical equity and hospital efficiency to determine the optimal referral rate across various regions of China, aiming to facilitate the establishment of an ordered health delivery system [8]. Equity is the basic principle of the ordered health delivery system. Given the constraints imposed by limited medical resources, to ensure equitable access to medical services for all individuals, it becomes essential for medical institutions at all levels to adjust the severity of cases they handle according to their respective service capacities [43]. The construction of the ordered health delivery system should also be aimed at achieving optimal utilization, ensuring that each medical institution allocates resources to where they are most required. Medical institutions need to adjust their service scope based on their functional positioning, leveraging their unique advantages, and maximizing the benefits derived from resource consumption [17]. Therefore, this study examines the effects of DIP reform on constructing the ordered health delivery system, focusing on two dimensions: the severity of the case and the service scope of medical institutions.

#### Severity of the case

The severity of cases treated by medical institutions is assessed using three outcome variables: the proportion of patients receiving complicated procedures (PRCP), the average case mix index (CMI), and the proportion of primary-DIP-groups cases (PPC). PRCP is calculated as the number of patients who received at least one grade 3 or grade 4 surgery divided by the number of patients who received at least one surgery during the same period. Grade 3 and Grade 4 surgeries are characterized by high risk, complexity, and difficulty, making them suitable to be conducted in superior hospitals [44]. CMI refers to the average weight assigned to discharged patients from a medical institution, equivalent to one-thousandth of the average point volume of cases. It serves as an indicator to gauge the severity of cases. A higher CMI indicates more difficult and critical cases. PPC is calculated as the number of discharged patients in the primary DIP group divided by the number of discharged patients in the same period.

#### Service scope of medical institutions

Primary-DIP-groups coverage (PC) and non-primary-DIP-groups coverage (NPC) are used to evaluate the service scope of medical institutions. PC is calculated as the number of primary DIP groups covered by the medical institution divided by the total number of primary DIP groups in the region (the number of primary DIP groups in Tai’an is 150); NPC is calculated as the number of non-primary DIP groups covered by the medical institution divided by the total number of non-primary DIP groups in the region (the number of non-primary DIP groups in Tai’an is 4230).

### Statistical analysis

We use ITSA to assess the longitudinal effects of DIP payment reforms on outcome variables. ITSA has strong internal validity and is a powerful quasi-experimental design [45]. The standard ITSA regression model assumes the following form:

3 $$Y_{t} = \beta_{0} + \beta_{1} T_{t} + \beta_{2} X_{t} + \beta_{3} X_{t} T_{t} + \varepsilon_{t}$$

where $Y_{t}$ is the outcome variable measured at each time interval $t$; $T_{t}$ is the time since the beginning of the study; $X_{t}$ is a dummy variable representing the intervention, which is 0 before intervention and 1 after intervention; $X_{t} T$ is an interaction term; $\beta_{0}$ represents the baseline level of the outcome variable; $\beta_{1}$ is the slope of the outcome variable before the DIP reform; $\beta_{2}$ is the level change of the outcome variable after the intervention, and $\beta_{3}$ represents the slope change of the outcome variable after the intervention. We use the Durbin-Watson statistic to test for serial autocorrelation in the regression model and the ordinary least squares regression with Newey–West standard errors to handle autocorrelation (Table S9 in Additional file 4). All statistical analyses were performed using Stata/MP 17.0.

### Sensitivity checks

To check the robustness of the findings and the validity of the conclusions, we performed four sets of sensitivity checks. First, we exclude data from December 2022 and January 2023. Because China abandoned its three-year COVID-19 clearance policy on December 7, 2022, most people in China were infected with the COVID-19 epidemic within two months. Medical institutions exerted their utmost effort to manage this surge of respiratory infections, resulting in the inability to sustain regular operations [46]. Second, to test whether the DIP payment reform really had an effect, we set up four false intervention dates (three months and six months before and after the real implementation time) to fit the model to check whether the outcome variables increased or decreased immediately. Third, we identified specific DIP groups that exhibited significantly higher or lower point volumes than the average across all DIP groups. These extreme values might have altered the initial trend of the average CMI for medical institutions. Therefore, we utilize the proportion of cases with point volumes below 500 and exceeding 2000 as two alternative outcome variables for CMI. Finally, we removed data from cancer hospitals and psychiatric hospitals. Although some cancer hospitals and psychiatric hospitals also treat patients with general diseases and accept DIP payments, their service capabilities for general diseases are limited, thus not accurately portraying the overall capability of the hospital.

## Results

### Descriptive statistics

Patient characteristics and outcome variables in the sample are summarized in Table 1. The final sample includes 794,191 discharge cases before the DIP reform and 1,014,112 discharge cases after the DIP reform. The number of discharged cases increased after the reform, the proportion of patients treated in primary healthcare facilities increased, and the proportion of patients treated in tertiary and secondary hospitals decreased. In the entire sample, PRCP, CMI, and PPC decreased slightly, while PC and NPC increased, similar to the change patterns of outcome variables in primary healthcare facilities. Among different levels of medical institutions, primary healthcare facilities exhibit the lowest levels of PRCP, CMI, and NPC, while maintaining the highest PPC before and after the reform. After the reform, the PC of primary healthcare facilities surpassed that of tertiary hospitals but was lower than that of secondary hospitals.

**Table 1:** Descriptive statistics of discharged patient characteristics and outcome variables before and after implementation of DIP reform

Variables	Before DIP reform (2020.4–2021.9)	After DIP reform (2021.10–2023.3)
Patient characteristics		
Discharge cases, No	794191	1,014112
Hospital level, No. (%)		
Tertiary	287773 (36.23)	325787 (32.13)
Secondary	337562 (42.50)	408704 (40.30)
Primary	168856 (21.26)	279621 (27.57)
Male sex, No. (%)	408504 (51.44)	518981 (51.18)
Age, mean (SD)	55.52 (20.27)	56.18 (21.11)
Insurance type		
UEBMI, No. (%)	241605 (30.42)	302732 (29.85)
URBMI, No. (%)	552586 (69.58)	711380 (70.15)
Outcome variables		
All patients		
PRCP (%)	15.99	15.65
CMI	1.16	1.15
PPC (%)	16.61	15.35
PC (%)	46.47	65.81
NPC (%)	19.26	27.09
Hospital level: Tertiary		
PRCP (%)	25.23	25.23
CMI	1.74	1.83
PPC (%)	4.98	4.63
PC (%)	52.05	51.29
NPC (%)	50.65	50.27
Hospital level: Secondary		
PRCP (%)	13.20	15.12
CMI	0.99	1.03
PPC (%)	21.39	17.94
PC (%)	72.77	73.11
NPC (%)	45.42	47.84
Hospital level: Primary		
PRCP (%)	5.82	5.26
CMI	0.52	0.54
PPC (%)	26.89	24.04
PC (%)	37.33	64.77
NPC (%)	7.74	18.12

### Impact of DIP payment reform on tertiary hospitals

**Fig. 2:**
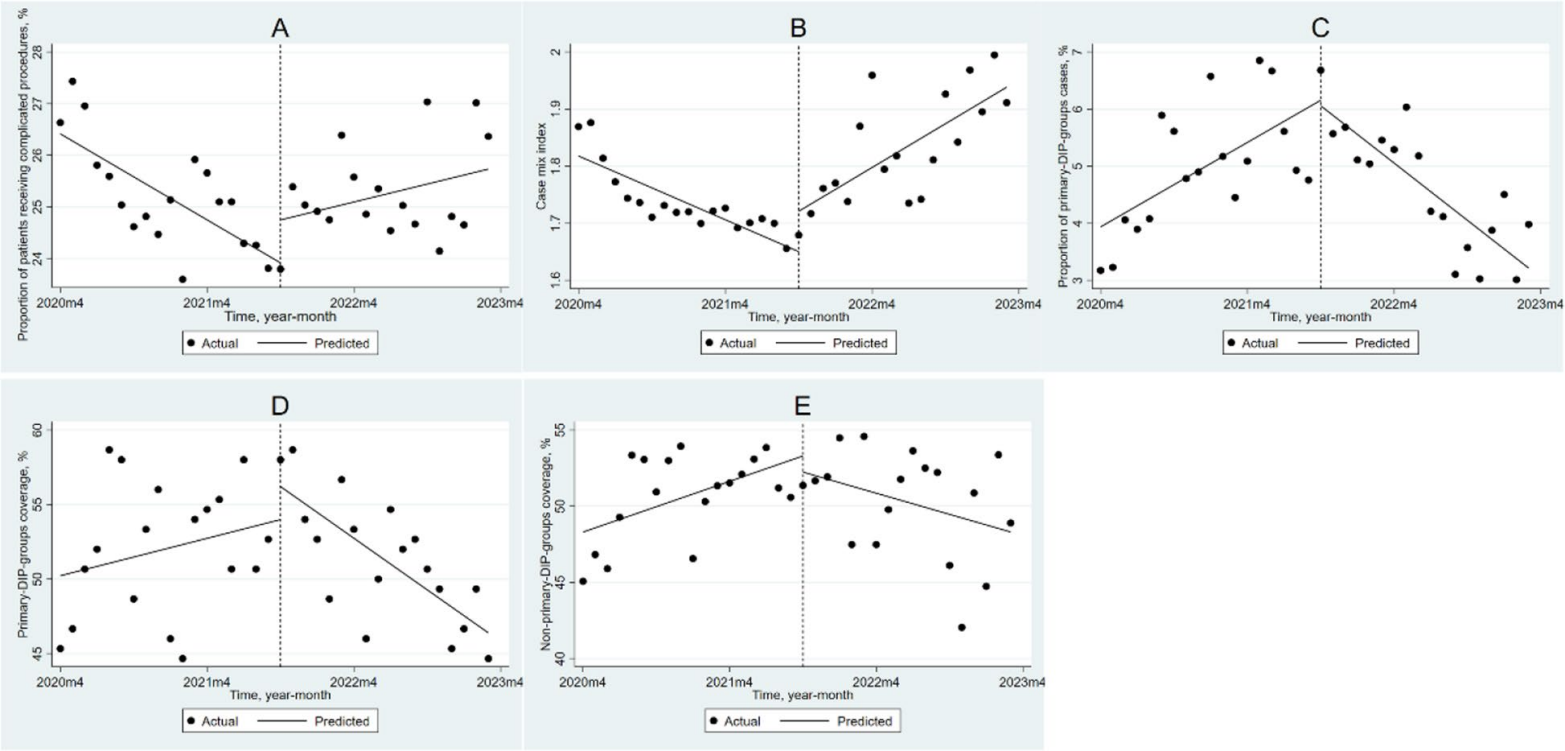
Changes in outcome variables in tertiary hospitals after implementing DIP payments using ITSA. **A**: the proportion of patients receiving complicated procedures; (**B**): case mix index; (**C**): the proportion of primary-DIP-groups cases; (**D**): primary-DIP-groups coverage; (**E**): non-primary-DIP-groups coverage

Table 2 and Fig. 2 show the results of ITSA for tertiary hospitals. Before the implementation of DIP, both PRCP (β_1_ = -0.139, *P* < 0.001) and CMI (β_1_ = -0.009, *P* < 0.001) in tertiary hospitals showed a downward trend. However, the DIP reform caused the monthly trend of PRCP to increase by 0.197% (*P* < 0.001), the immediate level of CMI to increase by 0.071 (*P* = 0.018), and the monthly trend of CMI to increase by 0.022 (*P* < 0.001). Before the DIP reform, tertiary hospitals exhibited an increasing trend in PPC (β_1_ = 0.123, *P* = 0.006) and NPC (β_1_ = 0.278, *P* = 0.015), while the monthly trend of PC was not significant. After the implementation of DIP, the monthly trends of PPC, PC, and NPC decreased significantly by 0.290% (*P* < 0.001), 0.786% (*P* < 0.001), and 0.509% (*P* = 0.008), respectively.

**Table 2:** The impact of DIP payment reform on different levels of medical institutions based on ITSA

Hospital-Level	Variables	Baseline slope β_1_ (95%CI)	Step change β_2_ (95%CI)	Slope change β_3_ (95%CI)
Tertiary	PRCP	-0.139 (-0.196, -0.082)***	0.832 (-0.072, 1.736)	0.197 (0.094, 0.300)***
	CMI	-0.009 (-0.013, -0.006)***	0.071 (0.013, 0.128)*	0.022 (0.016, 0.029)***
	PPC	0.123 (0.038, 0.208)**	-0.097 (-1.208, 1.014)	-0.290 (-0.392, -0.188)***
	PC	0.209 (-0.146, 0.564)	2.216 (-2.205, 6.637)	-0.786 (-1.207, -0.366)**
	NPC	0.278 (0.057, 0.498)*	-1.059 (-4.005, 1.888)	-0.509 (-0.873, -0.144)**
Secondary	PRCP	0.030 (-0.054. 0.114)	0.291 (-0.874, 1.456)	0.132 (0.022, 0.242)*
	CMI	-0.004 (-0.005, -0.002)***	0.037 (0.015, 0.059)**	0.008 (0.006, 0.010)***
	PPC	-0.010 (-0.190, 0.169)	6.940 (3.684, 10.196)***	-1.200 (-1.533, -0.878)***
	PC	-0.935 (-1.349, -0.521)***	14.982 (9.819, 20.144)***	0.257 (-0.330, 0.843)
	NPC	0.042 (-0.180, 0.264)	-4.058 (-7.343, -0.773)*	0.672 (0.379, 0.966)***
Primary	PRCP	-0.026 (-0.104, 0.051)	-1.676 (-2.736, -0.615)**	0.186 (0.078, 0.294)**
	CMI	-0.001 (-0.002, -0.001)***	0.019 (0.009, 0.028)***	0.002 (0.001, 0.003)***
	PPC	-0.133 (-0.251, -0.015)*	3.926 (0.285, 7.568)*	-0.515 (-0.864, -0.166)**
	PC	2.294 (1.545, 3.042)***	3.250 (-5.654, 12.155)	-2.011 (-2.892, -1.130)***
	NPC	0.657 (0.506, 0.809)***	-0.387 (-2.145, 1.370)	-0.124 (-0.297, 0.049)

*PRCP* Proportion of patients receiving complicated procedures, *CMI* Case mix index, *PPC* Proportion of primary-DIP-groups cases, *PC* Primary-DIP-groups coverage, *NPC* Non-primary-DIP-groups coverage

*$$p < 0.05$$

**$$p < 0.01$$

***$$p < 0.001$$

### Impact of DIP payment reform on secondary hospitals

**Fig. 3:**
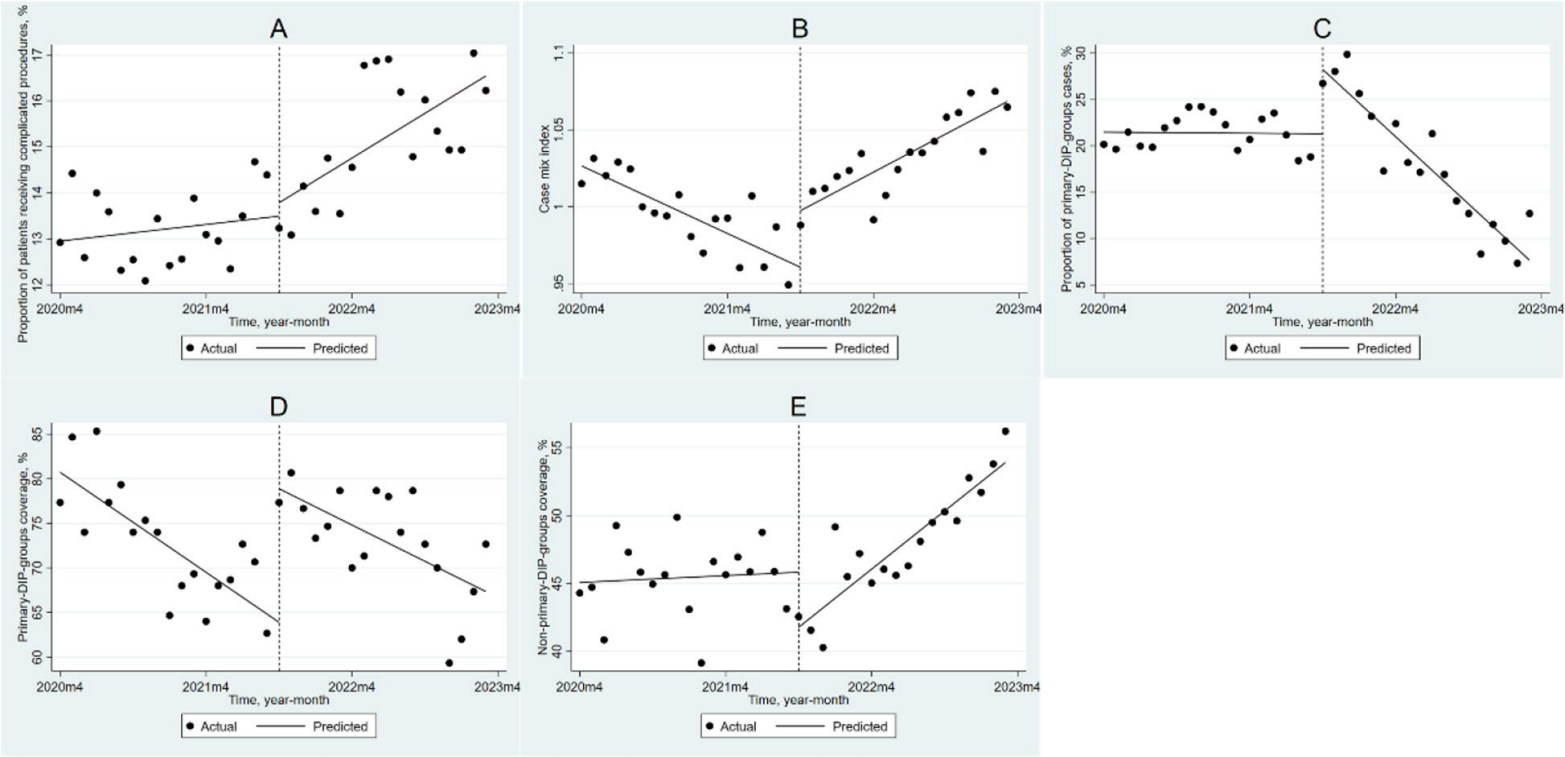
Changes in outcome variables in secondary hospitals after implementing DIP payments using ITSA. **A**: the proportion of patients receiving complicated procedures; (**B**): case mix index; (**C**): the proportion of primary-DIP-groups cases; (**D**): primary-DIP-groups coverage; (E): non-primary-DIP-groups coverage

For secondary hospitals (Table 2, Fig. 3), similar to tertiary hospitals, the PRCP monthly trend increased by 0.132% (*P* = 0.020) after the DIP reform; the CMI increased by 0.037 (*P* = 0.002) immediately in the month of reform, and the monthly trend increased by 0.008 (*P* < 0.001) after the reform. Additionally, the implementation of DIP resulted in an immediate increase of 6.940% (*P* < 0.001) and a monthly trend decrease of 1.200% (*P* < 0.001) in PPC. PC decreased by 0.935% (*P* < 0.001) per month before the DIP reform and immediately increased by 14.982% (*P* < 0.001) in the month of the DIP reform. NPC decreased by 4.058% (*P* = 0.017) in the month of reform, but the monthly trend increased by 0.672% (*P* < 0.001) after the reform.

### Impact of DIP payment reform on primary healthcare facilities

**Fig. 4:**
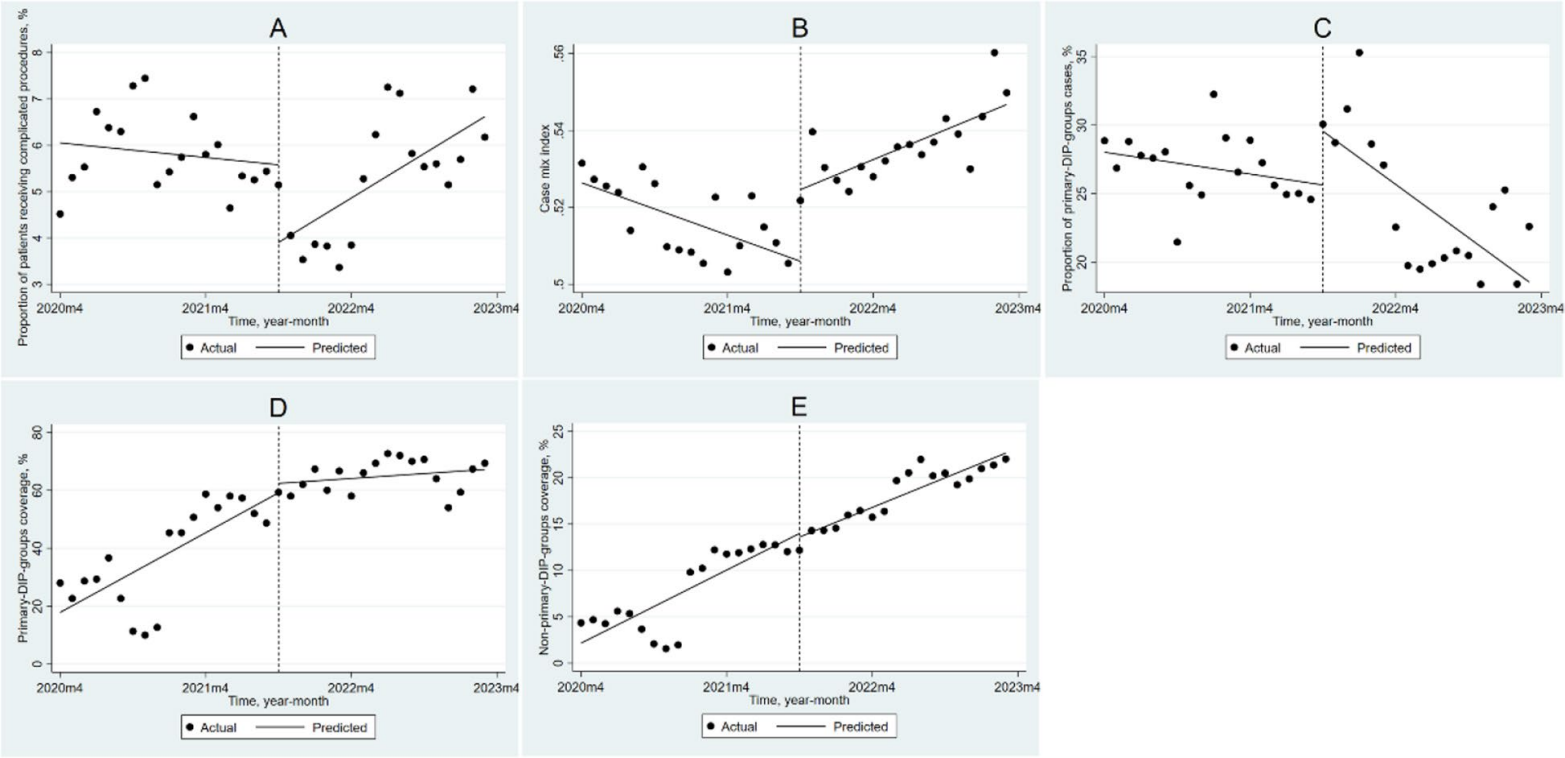
Changes in outcome variables in primary healthcare facilities after implementing DIP payments using ITSA. **A**: the proportion of patients receiving complicated procedures; (**B**): case mix index; (**C**): the proportion of primary-DIP-groups cases; (**D**): primary-DIP-groups coverage; (**E**): non-primary-DIP-groups coverage

For primary healthcare facilities (Table 2, Fig. 4), PRCP decreased by 1.676% (*P* = 0.003) in the month of reform, but the monthly trend increased by 0.186% (*P* < 0.001) after the reform. The CMI was decreasing by 0.001 (*P* < 0.001) per month before the reform, but the reform caused the CMI to increase immediately by 0.019 (*P* < 0.001), and the monthly trend increased by 0.002 (*P* < 0.001). PPC showed a downward trend before the reform (β_1_ = -0.133, *P* = 0.028), and although it increased significantly by 3.936% (*P* = 0.035) in the month of the reform, the monthly trend after the reform decreased significantly by 0.515% (*P* = 0.005). PC showed an upward trend before the reform (β_1_ = 2.294, *P* < 0.001), while the monthly trend decreased significantly by 2.011% (*P* < 0.001) after the reform. NPC increased by 0.657% (*P* < 0.001) per month before the reform, and the trend did not change significantly after the reform (*P* = 0.153).

### Sensitivity checks

Table S2 in Additional file 2 presents the analysis results excluding cases from December 2022 and January 2023, which are consistent with the total sample results. It can be seen from Table S3-S6 in Additional file 2 that across the four false intervention dates, DIP implementation was not significantly associated with immediate changes in most outcome variables, or their changes were smaller than the impact of DIP implementation in October 2021. Table S7 in Additional file 2 reports that the proportion of cases with a point volume less than 500 in tertiary and secondary hospitals significantly decreased in the month of reform, and the monthly trend decreased significantly in all levels of medical institutions after the reform; the proportion of cases with a point volume more than 2,000 in tertiary hospitals and primary healthcare facilities increased significantly in the month of reform, and the monthly trend increased significantly in all levels of medical institutions after the reform, which are consistent with the changing trend of CMI. In addition, after excluding cases from oncology and psychiatric hospitals, the trend of the outcome variables did not change (Table S8 in Additional file 2).

## Discussion

As far as we know, we provide the first empirical evidence to understand the impact of the DIP payment reform on the construction of the ordered health delivery system in China. Our results show that the severity of cases and the scope of services at different levels of medical institutions changed significantly after implementing the DIP.

The PRCP and CMI of tertiary and secondary hospitals both increased significantly, while the PPC decreased significantly after the reform, which represents an increase in the severity of cases treated in tertiary and secondary hospitals. The implementation of DIP appears to have encouraged tertiary hospitals to undertake treatment tasks aligned with their designated functions. One objective of the new medical reform is to ensure that “serious diseases do not leave the county” [47]. In China, most county-level hospitals are secondary hospitals. Secondary hospitals have maintained a low utilization rate for a long time, while tertiary hospitals have consistently sustained high admission rates [48]. After implementing the DIP reform, secondary hospitals began handling severe cases, suggesting a potential relief in treatment pressure for tertiary hospitals. PPC of tertiary and secondary hospitals showed a downward trend after the DIP reform, which is consistent with our expectations. Since the MIRC of primary DIP groups is lower than that of tertiary and secondary hospitals, treating cases within primary DIP groups without adequate reimbursement often results in reduced revenue [49]. However, it is essential to note that superior hospitals might sometimes be inclined to handle more cases within primary DIP groups. During an interview, a manager from a tertiary hospital stated: “Our hospital underwent expansion this spring, resulting in numerous vacant beds. Currently, the hospital is open to admitting cases within primary DIP groups. Having patients allows us to generate profit, even if the numbers are not substantial.”

Regarding service scope, tertiary hospitals have reduced their PC after the reform. Unexpectedly, the NPC of tertiary hospitals also showed a downward trend after the implementation of DIP. In response to market competition, medical institutions will adapt their service scope to secure a larger market share [50, 51]. The hospital holds a competitive edge for certain diseases due to its advanced medical technology and top-tier medical professionals. With comparatively lower medical costs, treating these cases results in higher profits. Consequently, the hospital provides significant support and incentives for treating these diseases. However, the hospital lacks the medical prowess to control costs for other diseases, often resulting in reduced revenue or losses within a fixed point volume system. As a result, the hospital tends to abstain from offering services for these conditions. For secondary hospitals, it is encouraging that we observed a significant increase in PC in the month of reform, although there was still a downward trend after the reform. A manager of a secondary hospital pointed out: “After the implementation of DIP, we wanted to expand the scope of services, but then we found that we could not make a profit from it because our technology lagged behind tertiary hospitals and had no advantage in the competition.”

The implementation of DIP payment reform has brought a series of unexpected results to primary healthcare facilities. After the DIP reform, PRCP and CMI in primary healthcare facilities showed an upward trend, and PPC showed a downward trend. On the one hand, the increase in CMI represents the improvement of the service capabilities of primary healthcare facilities, which is consistent with the aim of the DIP payment reform. On the other hand, considering the changes in these three outcome variables collectively, the severity of cases treated by primary healthcare facilities has increased, which may indicate that primary healthcare facilities have the risk of gradually tending to receive severe cases that exceed their service capabilities. The growth of PRCP indicates that primary healthcare facilities have increased treatment intensity, with some cases performing minor surgery or conservative treatment switching to performing complex procedures. Studies have shown that primary healthcare facilities are suitable for performing minor surgeries or providing post-surgery recovery. However, the risk associated with mortality or postoperative complications arising from complex procedures is not manageable by these primary healthcare facilities, which may cause further public concern about the quality of healthcare [6, 52, 53]. Under the new payment method, providers were found to tend to code patients into higher reimbursement groups, i.e., upcoding. Classifying patients into higher severity is a common form of upcoding (Jürges and Köberlein 2015). Besides handling severe cases, upcoding could also significantly contribute to the increasing CMI and decreasing PPC in primary healthcare facilities. According to the head of the Tai’an Healthcare Security Administration: “The service capabilities of primary healthcare facilities in Tai’an are still quite restricted. Rather than assuming the risks associated with treating severe cases, they are more inclined toward upcoding due to the comparatively lower penalties involved.” Table S1 in the Additional file 1 demonstrates that primary DIP groups typically exhibit lower point volumes, with 134 out of 150 primary DIP groups having point volumes below 1,000. Under the current MIRC setting, even if the case is classified into the primary DIP group, primary healthcare facilities can only obtain approximately 1.1 times (0.5/0.45) of the original point volume. However, if the case is classified into a higher severity DIP group, there is the potential to receive several times the original point volume. The reform did not hinder primary healthcare facilities from broadening the service scope of non-primary DIP groups. Conversely, the expansion of the service scope for the primary DIP group stagnated after the reform. For primary medical institutions, it is a temptation and inappropriate behavior to expand the scope of services that are not consistent with their functional positioning for the sake of profit [6]. Furthermore, the reduction in the service scope of primary DIP groups may cause more patients with minor illnesses to flow into crowded superior hospitals, further diminishing medical resource utilization efficiency and increasing inequity [54].

From 2020 to 2022, China faced the fallout from the COVID-19 epidemic. It is worth noting that megacities with populations exceeding 10 million experienced significant impacts. Economic growth decelerated, and there was a deviation in the distribution of medical resources from the usual pattern [55]. In contrast to megacities, Tai’an experienced a smaller transient population, a slower rate of COVID-19 case growth, and did not enforce city-wide lockdowns during the epidemic [56]. Nevertheless, the impact of the COVID-19 epidemic on healthcare utilization patterns cannot be ignored. For example, during the epidemic, the inter-regional movement was restricted, preventing the transfer of some critical cases to superior hospitals in larger cities, which may lead to an increase in the severity of cases admitted to tertiary hospitals in Tai’an. In addition, during the severe months of the epidemic, some primary healthcare facilities were converted into fever clinics and stopped treating inpatients without fever symptoms. This shift in functions may lead to abnormal fluctuations in outcome variables within primary healthcare facilities. The COVID-19 epidemic in China ended in 2023. Therefore, longer-term observational data are needed in the future to exclude the impact of the COVID-19 epidemic on the healthcare system.

Overall, our results indicate that the DIP reform did not effectively promote the construction of the ordered health delivery system. Although tertiary and secondary hospitals have assumed more severe treatment tasks, primary healthcare facilities have also reduced the treatment of patients with minor illnesses and performed more complex procedures. Fig. S1 in Additional file 3 indicates the impact of DIP payment on the total point volume of medical institutions in Tai’an. Under the DIP payment, medical institutions are strongly incentivized to increase their total point volumes to occupy more medical insurance funds. Despite the DIP payment reform setting up primary DIP groups, more points were obtained for treating severe cases, prompting primary healthcare facilities to change their original strategies. Giving more financial subsidies to primary healthcare facilities may reduce their incentives to pursue high points blindly. However, it is worth noting that through interviews with heads of Jinhua and Xuzhou Healthcare Security Administrations, we learned that certain county governments in Xuzhou and Jinhua have implemented a “two lines of revenue and expenditure” policy for primary healthcare facilities. That is to say, the government provides complete funding for the expenses of primary healthcare facilities, and consequently, all revenue generated by these institutions is remitted to the government. Although this incentive measure reduces the motivation of primary healthcare facilities to treat severe cases and perform complex procedures, it also reduces their productivity [57]. Since 2015, certain provinces have discontinued this policy due to its associated drawbacks; however, there are still regions across the country where it remains in effect [1]. Another feasible solution is to modify the MIRC settings and expand the count of primary DIP groups. Setting different values for the MIRC of primary DIP groups across various hospital levels is an option. Primary healthcare facilities could have the highest MIRC among primary DIP groups, followed by secondary hospitals, with tertiary hospitals having the lowest. Increasing the number of primary DIP groups is a trend in the development of DIP. For example, the Zhuhai Healthcare Security Administration implements dynamic adjustments to primary DIP groups. With the improvement of the service capabilities of primary healthcare facilities, there are plans to augment the existing 185 primary DIP groups by an additional 123 groups in the future. In addition, establishing a strict regulatory regime is critical to regulating the behavior of medical providers. Despite regulatory systems being established in the cities we interviewed, identifying violations within DIP payments remains difficult, posing challenges to audits. According to the head of the Guangzhou Healthcare Security Administration: “If a medical institution is found to have violated regulations during the treatment of a certain case, the points of the case will not be counted and will be subtracted from annual payment for the medical institution at double the inpatient costs. However, numerous inappropriate behaviors leading to increased consumption of medical insurance funds still evade detection.”

## Limitations

This study has several limitations. First, due to the absence of an index system that is in accordance with specific national conditions of China for evaluating the construction of the ordered health delivery system through DIP reform, it remains uncertain whether the chosen outcome variables adequately assess the effects of DIP reform on this aspect. Therefore, further longitudinal observation and examination of additional practical cases are warranted to validate our findings. Second, the payment method reform is a nationwide policy, and throughout 2022, most cities across China have been gradually implementing DIP/DRG reform. Due to data availability, there is no suitable unaffected control group, which limits our ability to exclude confounding factors and external events. In addition, ITSA without a control group is only applicable for assessing the impact of reforms; however, it cannot provide robust evidence to support the causal relationship between intervention and outcome variables. Third, based solely on the information acquired during interviews, we discovered inappropriate behaviors, such as upcoding, in medical institutions. However, due to the absence of an efficient regulatory system, we lack direct evidence to quantify the number of upcoding cases influenced by the reform. Fourth, the empirical findings of this study should be treated with caution. Since there are considerable differences in economic development and medical conditions across China, payment reform in other cities may have different impacts.

## Conclusions

Although the design of the DIP payment system reflects the government’s determination to promote the construction of the ordered health delivery system, it cannot achieve the expected results under the current economic incentives. While tertiary and secondary hospitals have taken on more severe treatment tasks, primary healthcare facilities have also begun to treat fewer patients with minor illnesses and perform more complex procedures. Policymakers should change incentives and implement restraint mechanisms to regulate the behavior of medical institutions.

### Supplementary Information

**Supplementary Material 1.**

**Supplementary Material 2.**

**Supplementary Materia 3.**

**Supplementary Material 4.**

## Data Availability

No datasets were generated or analysed during the current study.
