# Supplementary material for: Does a new case-based payment system promote the construction of the ordered health delivery system? Evidence from a pilot city in China
Source: Int J Equity Health. 2024 Mar 14;23:55. doi: 10.1186/s12939-024-02146-y (PMC10938765; doi:10.1186/s12939-024-02146-y)
Supplement: Supplementary file 1 — Supplementary Material 1. [file 12939_2024_2146_MOESM1_ESM.docx]

**Table S1** 150 primary DIP groups set up by the Tai'an Healthcare Security Administration

| No. | ICD-10 codes | Principal diagnosis | ICD-9-CM-3 codes | Principal procedures | Point volume |
| --- | --- | --- | --- | --- | --- |
| 1 | A09.0 | Other and unspecified gastroenteritis and colitis of infectious origin |  | Conservative treatments | 272 |
| 2 | A09.9 | Gastroenteritis and colitis of unspecified origin |  | Conservative treatments | 464 |
| 3 | B00.2 | Herpesviral gingivostomatitis and pharyngotonsillitis |  | Conservative treatments | 354 |
| 4 | B01.9 | Varicella without complication |  | Conservative treatments | 316 |
| 5 | B08.5 | Enteroviral vesicular pharyngitis |  | Conservative treatments | 283 |
| 6 | D13.1 | Stomach |  | Conservative treatments | 1523 |
| 7 | D17.1 | Benign lipomatous neoplasm of skin and subcutaneous tissue of trunk | 34.4 | Excision or destruction of lesion of chest wall | 579 |
| 8 | D17.2 | Benign lipomatous neoplasm of skin and subcutaneous tissue of limbs | 82.29 | Excision of soft tissue lesion | 761 |
| 9 | D17.3 | Benign lipomatous neoplasm of skin and subcutaneous tissue of other and unspecified sites | 86.3 | Excision of lesion of skin and subcutaneous tissue | 485 |
| 10 | D17.9 | Benign lipomatous neoplasm, unspecified | 82.29 | Excision of soft tissue lesion | 532 |
| 11 | D23.4 | Skin of scalp and neck | 86.3 | Other local excision or destruction of lesion or tissue of skin and subcutaneous tissue | 732 |
| 12 | D25.9 | Leiomyoma of uterus, unspecified |  | Conservative treatments | 1206 |
| 13 | D50.9 | Iron deficiency anaemia, unspecified |  | Conservative treatments | 713 |
| 14 | D64.9 | Anaemia, unspecified |  | Conservative treatments | 673 |
| 15 | D69.0 | Allergic purpura |  | Conservative treatments | 470 |
| 16 | E03.9 | Hypothyroidism, unspecified |  | Conservative treatments | 716 |
| 17 | E04.1 | Nontoxic single thyroid nodule |  | Conservative treatments | 538 |
| 18 | E04.9 | Nontoxic goitre, unspecified |  | Conservative treatments | 1303 |
| 19 | E05.9 | Thyrotoxicosis, unspecified |  | Conservative treatments | 521 |
| 20 | E10.9 | Diabetes mellitus without complications |  | Conservative treatments | 649 |
| 21 | E11.6 | Non-insulin-dependent diabetes mellitus with other specified complications |  | Conservative treatments | 788 |
| 22 | E11.9 | Non-insulin-dependent diabetes mellitus without complications |  | Conservative treatments | 764 |
| 23 | E87.6 | Hypokalaemia |  | Conservative treatments | 574 |
| 24 | F03.x | Unspecified dementia |  | Conservative treatments | 1134 |
| 25 | G20.x | Parkinson's disease |  | Conservative treatments | 741 |
| 26 | G45.0 | Vertebro-basilar artery syndrome |  | Conservative treatments | 563 |
| 27 | G45.9 | Transient cerebral ischaemic attack, unspecified |  | Conservative treatments | 607 |
| 28 | G50.0 | Trigeminal neuralgia |  | Conservative treatments | 508 |
| 29 | H16.2 | Keratoconjunctivitis |  | Conservative treatments | 345 |
| 30 | H25.9 | Senile cataract, unspecified |  | Conservative treatments | 571 |
| 31 | H66.9 | Otitis media, unspecified |  | Conservative treatments | 363 |
| 32 | I09.9 | Rheumatic heart disease, unspecified |  | Conservative treatments | 706 |
| 33 | I10.x | Essential (primary) hypertension |  | Conservative treatments | 594 |
| 34 | I20.8 | Other forms of angina pectoris |  | Conservative treatments | 771 |
| 35 | I25.1 | Atherosclerotic heart disease |  | Conservative treatments | 667 |
| 36 | I27.9 | Pulmonary heart disease, unspecified |  | Conservative treatments | 914 |
| 37 | I67.9 | Cerebrovascular disease, unspecified |  | Conservative treatments | 633 |
| 38 | I83.9 | Varicose veins of lower extremities without ulcer or inflammation |  | Conservative treatments | 886 |
| 39 | I86.1 | Scrotal varices |  | Conservative treatments | 742 |
| 40 | J02.9 | Acute pharyngitis, unspecified |  | Conservative treatments | 473 |
| 41 | J03.9 | Acute tonsillitis, unspecified |  | Conservative treatments | 319 |
| 42 | J06.9 | Acute upper respiratory infection, unspecified |  | Conservative treatments | 309 |
| 43 | J12.9 | Viral pneumonia, unspecified |  | Conservative treatments | 934 |
| 44 | J15.7 | Pneumonia due to Mycoplasma pneumoniae |  | Conservative treatments | 384 |
| 45 | J15.9 | Bacterial pneumonia, unspecified |  | Conservative treatments | 847 |
| 46 | J16.0 | Chlamydial pneumonia |  | Conservative treatments | 463 |
| 47 | J18.0 | Bronchopneumonia, unspecified |  | Conservative treatments | 427 |
| 48 | J18.1 | Lobar pneumonia, unspecified |  | Conservative treatments | 711 |
| 49 | J20.9 | Acute bronchitis, unspecified |  | Conservative treatments | 417 |
| 50 | J31.2 | Chronic pharyngitis |  | Conservative treatments | 704 |
| 51 | J42.x | Unspecified chronic bronchitis |  | Conservative treatments | 600 |
| 52 | J43.9 | Emphysema, unspecified |  | Conservative treatments | 786 |
| 53 | J44.9 | Chronic obstructive pulmonary disease, unspecified |  | Conservative treatments | 729 |
| 54 | J45.0 | Predominantly allergic asthma |  | Conservative treatments | 558 |
| 55 | J45.9 | Asthma, unspecified |  | Conservative treatments | 696 |
| 56 | J93.9 | Pneumothorax, unspecified |  | Conservative treatments | 748 |
| 57 | K05.2 | Acute periodontitis |  | Conservative treatments | 390 |
| 58 | K12.2 | Cellulitis and abscess of mouth |  | Conservative treatments | 387 |
| 59 | K21.9 | Gastro-oesophageal reflux disease without oesophagitis |  | Conservative treatments | 624 |
| 60 | K22.8 | Other specified diseases of oesophagus |  | Conservative treatments | 942 |
| 61 | K25.9 | Gastric ulcer, unspecified as acute or chronic, without haemorrhage or perforation |  | Conservative treatments | 651 |
| 62 | K29.3 | Chronic superficial gastritis |  | Conservative treatments | 538 |
| 63 | K31.1 | Adult hypertrophic pyloric stenosis |  | Conservative treatments | 325 |
| 64 | K35.8 | Acute appendicitis, unspecified |  | Conservative treatments | 684 |
| 65 | K40.9 | Unilateral or unspecified inguinal hernia, without obstruction or gangrene |  | Conservative treatments | 850 |
| 66 | K52.9 | Noninfective gastroenteritis and colitis, unspecified |  | Conservative treatments | 371 |
| 67 | K61.0 | Anal abscess |  | Conservative treatments | 673 |
| 68 | K62.1 | Rectal polyp |  | Conservative treatments | 743 |
| 69 | K63.5 | Polyp of colon |  | Conservative treatments | 731 |
| 70 | K63.8 | Other specified diseases of intestine |  | Conservative treatments | 745 |
| 71 | K76.8 | Other specified diseases of liver |  | Conservative treatments | 719 |
| 72 | K80.2 | Calculus of gallbladder without cholecystitis |  | Conservative treatments | 620 |
| 73 | K80.5 | Calculus of bile duct without cholangitis or cholecystitis |  | Conservative treatments | 840 |
| 74 | K81.0 | Acute cholecystitis |  | Conservative treatments | 736 |
| 75 | K82.8 | Other specified diseases of gallbladder |  | Conservative treatments | 848 |
| 76 | K86.1 | Other chronic pancreatitis |  | Conservative treatments | 819 |
| 77 | K91.8 | Other postprocedural disorders of digestive system, not elsewhere classified |  | Conservative treatments | 550 |
| 78 | L04.0 | Acute lymphadenitis of face, head and neck |  | Conservative treatments | 363 |
| 79 | L04.9 | Acute lymphadenitis, unspecified |  | Conservative treatments | 470 |
| 80 | M06.9 | Rheumatoid arthritis, unspecified |  | Conservative treatments | 771 |
| 81 | M12.5 | Traumatic arthropathy |  | Conservative treatments | 736 |
| 82 | M30.0 | Polyarteritis nodosa |  | Conservative treatments | 854 |
| 83 | M67.4 | Ganglion |  | Conservative treatments | 433 |
| 84 | N03.9 | Chronic nephritic syndrome, unspecified |  | Conservative treatments | 702 |
| 85 | N39.0 | Urinary tract infection, site not specified |  | Conservative treatments | 557 |
| 86 | N40.x | Hyperplasia of prostate |  | Conservative treatments | 765 |
| 87 | N41.1 | Chronic prostatitis |  | Conservative treatments | 621 |
| 88 | N43.3 | Hydrocele, unspecified |  | Conservative treatments | 886 |
| 89 | N45.9 | Orchitis, epididymitis and epididymo-orchitis without abscess |  | Conservative treatments | 424 |
| 90 | N48.1 | Balanoposthitis |  | Conservative treatments | 386 |
| 91 | N61.x | Inflammatory disorders of breast |  | Conservative treatments | 427 |
| 92 | N64.9 | Disorder of breast, unspecified |  | Conservative treatments | 400 |
| 93 | N80.0 | Endometriosis of uterus |  | Conservative treatments | 1055 |
| 94 | N83.2 | Other and unspecified ovarian cysts |  | Conservative treatments | 897 |
| 95 | N85.8 | Other specified noninflammatory disorders of uterus |  | Conservative treatments | 527 |
| 96 | N88.8 | Other specified noninflammatory disorders of cervix uteri |  | Conservative treatments | 338 |
| 97 | N89.8 | Other specified noninflammatory disorders of vagina |  | Conservative treatments | 468 |
| 98 | N90.7 | VuIvar cyst |  | Conservative treatments | 483 |
| 99 | N90.8 | Other specified noninflammatory disorders of vulva and perineum |  | Conservative treatments | 369 |
| 100 | N90.9 | Noninflammatory disorder of vulva and perineum, unspecified |  | Conservative treatments | 419 |
| 101 | N92.6 | Irregular menstruation, unspecified |  | Conservative treatments | 797 |
| 102 | N93.8 | Other specified abnormal uterine and vaginal bleedlng |  | Conservative treatments | 548 |
| 103 | O21.0 | Mild hyperemesis gravidarum |  | Conservative treatments | 232 |
| 104 | O24.9 | Diabetes mellitus in pregnancy, unspecified |  | Conservative treatments | 364 |
| 105 | O86.8 | Other specified puerperal infections |  | Conservative treatments | 324 |
| 106 | R22.0 | Localized swelling, mass and lump, head |  | Conservative treatments | 436 |
| 107 | R22.1 | Localized swelling, mass and lump, neck |  | Conservative treatments | 418 |
| 108 | R22.2 | Localized swelling, mass and lump, trunk |  | Conservative treatments | 451 |
| 109 | R22.3 | Localized swelling, mass and lump, upper limb |  | Conservative treatments | 368 |
| 110 | R22.4 | Localized swelling, mass and lump, lower limb |  | Conservative treatments | 374 |
| 111 | S00.8 | Superficial injury of other parts of head |  | Conservative treatments | 385 |
| 112 | S00.9 | Superficial injury of head, part unspecified |  | Conservative treatments | 445 |
| 113 | S01.0 | Open wound of scalp |  | Conservative treatments | 413 |
| 114 | S01.8 | Open wound of other parts of head | 86.28 | Nonexcisional debridement of wound, infection or burn | 743 |
| 115 | S33.5 | Sprain and strain of lumbar spine |  | Conservative treatments | 585 |
| 116 | S42.0 | Fracture of clavicle |  | Conservative treatments | 955 |
| 117 | S42.2 | Fracture of upper end of humerus |  | Conservative treatments | 1166 |
| 118 | S42.3 | Fracture of shaft of humerus |  | Conservative treatments | 1059 |
| 119 | S43.1 | Dislocation of acromioclavicular joint |  | Conservative treatments | 1400 |
| 120 | S52.2 | Fracture of shaft of ulna |  | Conservative treatments | 577 |
| 121 | S52.4 | Fracture of shafts of both ulna and radius |  | Conservative treatments | 715 |
| 122 | S60.2 | Contusion of other parts of wrist and hand | 86.59 | Sutures | 544 |
| 123 | S61.1 | Open wound of finger(s) with damage to nail |  | Conservative treatments | 598 |
| 124 | S62.3 | Fracture of other metacarpal bone |  | Conservative treatments | 701 |
| 125 | S62.6 | Fracture of other finger |  | Conservative treatments | 704 |
| 126 | S72.1 | Pertrochanteric fracture |  | Conservative treatments | 1419 |
| 127 | S72.3 | Fracture of shaft of femur |  | Conservative treatments | 1417 |
| 128 | S80.0 | Contusion of knee |  | Conservative treatments | 436 |
| 129 | S80.1 | Contusion of other and unspecified parts of lower leg |  | Conservative treatments | 540 |
| 130 | S81.9 | Open wound of lower leg, part unspecified |  | Conservative treatments | 607 |
| 131 | S82.0 | Fracture of patella |  | Conservative treatments | 1184 |
| 132 | S82.1 | Fracture of upper end of tibia |  | Conservative treatments | 643 |
| 133 | S82.2 | Fracture of shaft of tibia |  | Conservative treatments | 1338 |
| 134 | S82.3 | Fracture of lower end of tibia |  | Conservative treatments | 2216 |
| 135 | S82.5 | Fracture of medial malleolus |  | Conservative treatments | 1095 |
| 136 | S82.6 | Fracture of lateral malleolus |  | Conservative treatments | 777 |
| 137 | S82.8 | Fractures of other parts of lower leg |  | Conservative treatments | 1620 |
| 138 | S92.0 | Fracture of calcaneus |  | Conservative treatments | 798 |
| 139 | S92.3 | Fracture of metatarsal bone |  | Conservative treatments | 631 |
| 140 | S92.4 | Fracture of great toe |  | Conservative treatments | 562 |
| 141 | S93.4 | Sprain and strain of ankle |  | Conservative treatments | 434 |
| 142 | T00.9 | Multiple superficial injuries, unspecified |  | Conservative treatments | 509 |
| 143 | T01.9 | Multiple open wounds, unspecified |  | Conservative treatments | 344 |
| 144 | T13.1 | Open wound of lower limb, level unspecified |  | Conservative treatments | 1051 |
| 145 | T13.9 | Unspecified injury of lower limb, level unspecified |  | Conservative treatments | 455 |
| 146 | T14.0 | Superficial injury of unspecified body region |  | Conservative treatments | 448 |
| 147 | T14.1 | Open wound of unspecified body region |  | Conservative treatments | 443 |
| 148 | T14.2 | Fracture of unspecified body region |  | Conservative treatments | 515 |
| 149 | T79.3 | Post-traumatic wound infection, not elsewhere classified |  | Conservative treatments | 423 |
| 150 | Z54.0 | Convalescence following surgery |  | Conservative treatments | 617 |
