# Supplementary material for: Does a new case-based payment system promote the construction of the ordered health delivery system? Evidence from a pilot city in China
Source: Int J Equity Health. 2024 Mar 14;23:55. doi: 10.1186/s12939-024-02146-y (PMC10938765; doi:10.1186/s12939-024-02146-y)
Supplement: Supplementary file 2 — Supplementary Material 2. [file 12939_2024_2146_MOESM2_ESM.docx]

**Table S2** Robustness checks: exclude samples from December 2022 and January 2023

| Hospital-Level | Variables | Baseline slope β_1_ (95%CI) | Step change β_2_ (95%CI) | Slope change β_3_ (95%CI) |
| --- | --- | --- | --- | --- |
| Tertiary | PRCP | -0.139 (-0.197, -0.081)*** | 0.738 (-0.191, 1.667) | 0.225 (0.106, 0.345)** |
|  | CMI | -0.009 (-0.013, -0.006)*** | 0.072 (0.013, 0.130)* | 0.022 (0.015, 0.029)*** |
|  | PPC | 0.123 (0.038, 0.208)** | 0.093 (-1.029, 1.216) | -0.331 (-0.436, -0.226)*** |
|  | PC | 0.209 (-0.148, 0.566) | 2.141 (-2.388, 6.691) | -0.765 (-1.216, -0.314)** |
|  | NPC | 0.278 (0.055, 0.500)* | -0.998 (-4.058, 2.061) | -0.506 (-0.934, -0.079)* |
| Secondary | PRCP | 0.030 (-0.055, 0.115) | 0.023 (-1.076, 1.123) | 0.193 (0.081, 0.305)** |
|  | CMI | -0.004 (-0.005, -0.002)*** | 0.035 (0.013, 0.057)** | 0.008 (0.007, 0.010)*** |
|  | PPC | -0.010 (-0.191, 0.171) | 7.524 (4.200, 10.849)*** | -1.318 (-1.678, -0.957)*** |
|  | PC | -0.935 (-1.351, -0.518)*** | 13.933 (8.909, 18.957)*** | 0.513 (-0.007, 1.033) |
|  | NPC | 0.042 (-0.181, 0.265) | -4.192 (-7.650, -0.734)* | 0.712 (0.364, 1.061)*** |
| Primary | PRCP | -0.026 (-0.104, 0.052) | -1.863 (-3.001, -0.726)** | 0.230 (0.105, 0.354)** |
|  | CMI | -0.001 (-0.002, -0.001)*** | 0.018 (0.008, 0.028)*** | 0.003 (0.002, 0.004)*** |
|  | PPC | -0.133 (-0.251, -0.014)* | 5.154 (1.588, 8.721)** | -0.775 (-1.149, -0.402)*** |
|  | PC | 2.294 (1.541, 3.047)*** | 1.513 (-7.202, 10.228) | -1.607 (-2.438, -0.775)*** |
|  | NPC | 0.657 (0.505, 0.810)*** | -0.801 (-2.514, 0.912) | -0.037 (-0.222, 0.149) |

Note: *$p<0.05$, **$p<0.01$, ***$p<0.001$.

**Table S3** Robustness checks: false intervention dates (April 2022)

| Hospital-Level | Variables | Baseline slope β_1_ (95%CI) | Step change β_2_ (95%CI) | Slope change β_3_ (95%CI) |
| --- | --- | --- | --- | --- |
| Tertiary | PRCP | -0.063 (-0.127, 0.001) | 0.466 (-0.655, 1.587) | 0.148 (-0.004, 0.299) |
|  | CMI | -0.003 (-0.008, 0.002) | 0.091 (-0.043, 0.226) | 0.015 (-0.001, 0.031) |
|  | PPC | 0.077 (0.026, 0.127)** | -0.958 (-1.976, 0.059) | -0.254 (-0.384, -0.125)*** |
|  | PC | 0.202 (-0.051, 0.455) | -2.869 (-7.908, 2.169) | -0.710 (-1.278, -0.142)* |
|  | NPC | 0.179 (-0.002, 0.359) | -2.746 (-6.506, 1.015) | -0.362 (-0.919, 0.195) |
| Secondary | PRCP | 0.040 (-0.007, 0.087) | **2.123 (0.756, 3.490)**** | -0.054 (-0.240, 0.133) |
|  | CMI | -0.001 (-0.002, 0.001) | 0.014 (-0.014, 0.042) | 0.007 (0.004, 0.010)*** |
|  | PPC | 0.142 (-0.069, 0.353) | -3.349 (-7.853, 1.156) | -1.304 (-1.833, -0.775)*** |
|  | PC | -0.207 (-0.561, 0.146) | 4.520 (-3.096, 12.136) | -0.613 (-1.614, 0.388) |
|  | NPC | -0.013 (-0.189, 0.163) | -0.683 (-3.450, 2.085) | 0.969 (0.726, 1.211)*** |
| Primary | PRCP | -0.103 (-0.166, -0.040)** | 1.371(-0.113, 2.855) | 0.186 (0.002, 0.370)* |
|  | CMI | 0.000(-0.001, 0.001) | 0.008 (-0.002, 0.017) | 0.002 (0.001, 0.003)** |
|  | PPC | 0.093 (-0.078, -0.263) | **-8.717 (-11.994, -5.349)***** | 0.059 (-0.366, 0.485) |
|  | PC | 2.156 (1.688, 2.624)*** | -3.488 (-11.660, 4.685) | -2.331 (-3.479, -1.182)*** |
|  | NPC | 0.612 (0.515, 0.710)*** | 0.608 (-1.541, 2.757) | -0.220 (-0.475, 0.034) |

Note: *$p<0.05$, **$p<0.01$, ***$p<0.001$. The outcome variables highlighted in bold exhibited significant changes during the month of DIP reform, with a change range surpassing the actual change range observed in October 2021.

**Table S4** Robustness checks: false intervention dates (January 2022)

| Hospital-Level | Variables | Baseline slope β_1_ (95%CI) | Step change β_2_ (95%CI) | Slope change β_3_ (95%CI) |
| --- | --- | --- | --- | --- |
| Tertiary | PRCP | -0.103 (-0.166, -0.041)** | 0.976 (-0.048, 2.001) | 0.151 (0.025, 0.277)* |
|  | CMI | -0.006 (-0.011, -0.002)** | **0.109 (0.021, 0.196)*** | 0.018 (0.009, 0.026)*** |
|  | PPC | 0.111 (0.053, 0.169)*** | -0.884 (-1.812, 0.044) | -0.267 (-0.360, -0.175)*** |
|  | PC | 0.298 (0.024, 0.572)* | -2.680 (-7.216, 1.855) | -0.743 (-1.186, -0.300)** |
|  | NPC | 0.210 (0.036, 0.384)* | -1.234 (-4.887, 2.418) | -0.479 (-0.956, -0.002)* |
| Secondary | PRCP | 0.030 (-0.029, 0.089) | 1.110 (-0.199, 2.420) | 0.086 (-0.044, 0.216) |
|  | CMI | -0.002 (-0.004, 0.000)* | 0.034 (0.005, 0.064)* | 0.006 (0.004, 0.019)*** |
|  | PPC | 0.237 (0.004, 0.469)* | -1.261 (-5.711, 3.188) | -1.353 (-1.774, -0.931)*** |
|  | PC | -0.398 (-0.891, 0.095) | 7.664 (-0.120, 15.449) | -0.280 (-1.044, 0.484) |
|  | NPC | -0.116 (-0.320, 0.088) | 1.133 (-2.525, 4.792) | 0.744 (0.381, 1.107)*** |
| Primary | PRCP | -0.074 (-0.152, 0.004) | -0.609 (-1.816, 0.597) | 0.258 (0.139, 0.377)*** |
|  | CMI | 0.000 (-0.001, 0.001) | 0.008 (-0.005, 0.020) | 0.002 (0.001, 0.003)** |
|  | PPC | 0.027 (-0.147, 0.200) | -0.900 (-6.682, 4.882) | -0.579 (-1.196, 0.038) |
|  | PC | 2.234 (1.684,2.783)*** | 0.683 (-6.418, 7.785) | -2.236 (-3.067, -1.405)*** |
|  | NPC | 0.622 (0.505, 0.739)*** | 0.272 (-1.328, 1.872) | -0.144 (-0.300, 0.011) |

Note: *$p<0.05$, **$p<0.01$, ***$p<0.001$. The outcome variables highlighted in bold exhibited significant changes during the month of DIP reform, with a change range surpassing the actual change range observed in October 2021.

**Table S5** Robustness checks: false intervention dates (July 2021)

| Hospital-Level | Variables | Baseline slope β_1_ (95%CI) | Step change β_2_ (95%CI) | Slope change β_3_ (95%CI) |
| --- | --- | --- | --- | --- |
| Tertiary | PRCP | -0.131 (-0.226, -0.036)** | -0.079 (-1.137, 0.980) | 0.206 (0.093, 0.320)** |
|  | CMI | -0.011 (-0.015, -0.007)*** | 0.019 (-0.030, 0.069) | 0.024 (0.018, 0.030)*** |
|  | PPC | 0.205 (0.128, 0.282)*** | -0.676 (-1.733, 0.380) | -0.328 (-0.421, -0.235)*** |
|  | PC | 0.210 (-0.261, 0.680) | 2.902 (-2.183, 7.986) | -0.667 (-1.184, -0.149)* |
|  | NPC | 0.375 (0.138, 0.611)** | -0.857 (-3.437, 1.724) | -0.581 (-0.905, -0.257)** |
| Secondary | PRCP | -0.046 (-0.127, 0.035) | **1.002 (0.077, 1.927)*** | 0.183 (0.084, 0.282)** |
|  | CMI | -0.003 (-0.006, -0.001)** | 0.000 (-0.028, 0.027) | 0.008 (0.006, 0.011)*** |
|  | PPC | 0.177 (0.030, 0.325)* | 3.144 (-1.856, 8.145) | -0.995 (-1.370, -0.620)*** |
|  | PC | -1.186 (-1.696, -0.676)*** | 11.202 (4.722, 17.681)** | 0.902 (0.215, 1.589)* |
|  | NPC | 0.045 (-0.231, 0.321) | -3.218 (-7.377, -0.941) | 0.464 (0.096, 0.833)* |
| Primary | PRCP | 0.001 (-0.113, 0.115) | -1.638 (-2.931, -0.345)* | 0.097 (-0.034, 0.227) |
|  | CMI | -0.001 (-0.002, 0.000)* | 0.008 (0.008, 0.028) | 0.003 (0.002, 0.004)*** |
|  | PPC | -0.031 (-0.206, 0.144) | 1.462 (-2.604, 5.529) | -0.405 (-0.720, -0.090)* |
|  | PC | 2.521 (1.502, 3.541)*** | 2.696 (-8.703, 14.095) | -1.930 (-3.041, -0.819)** |
|  | NPC | 0.679 (0.460, 0.897)*** | -0.193 (-2.590, 2.204) | -0.148 (-0.377, 0.081) |

Note: *$p<0.05$, **$p<0.01$, ***$p<0.001$. The outcome variables highlighted in bold exhibited significant changes during the month of DIP reform, with a change range surpassing the actual change range observed in October 2021.

**Table S6** Robustness checks: false intervention dates (April 2021)

| Hospital-Level | Variables | Baseline slope β_1_ (95%CI) | Step change β_2_ (95%CI) | Slope change β_3_ (95%CI) |
| --- | --- | --- | --- | --- |
| Tertiary | PRCP | -0.226 (-0.396, -0.056)* | 0.565 (-0.970, 2.100) | 0.270 (0.091, 0.450)** |
|  | CMI | -0.015 (-0.020, -0.009)*** | 0.001 (-0.051, 0.053) | 0.026 (0.020, 0.033)*** |
|  | PPC | 0.196 (0.058, 0.334)** | 0.366 (-1.006, 1.739) | -0.317 (-0.463, -0.172)*** |
|  | PC | 0.138 (-0.679, 0.954) | 3.988 (-2.856, 10.833) | -0.499 (-1.333, 0.335) |
|  | NPC | 0.437 (0.014, 0.859)* | 0.031 (-3.690, 3.752) | -0.621 (-1.079, -0.163)** |
| Secondary | PRCP | -0.050 (-0.190, 0.089) | 0.257 (-0.889, 1.403) | 0.203 (0.056, 0.350)** |
|  | CMI | -0.005 (-0.007, -0.002)*** | -0.007 (-0.031, 0.017) | 0.009 (0.006, 0.011)*** |
|  | PPC | 0.234 (-0.085, 0.553) | 3.127 (-1.545, 7.800) | -0.894 (-1.293, -0.496)*** |
|  | PC | -1.298 (-2.025, -0.572)** | 5.319 (-1.133, 11.771) | 1.265 (0.443, 2.087)** |
|  | NPC | -0.041 (-0.597, 0.515) | -1.843 (-7.100, 3.413) | 0.421 (-0.163, 1.006) |
| Primary | PRCP | 0.079 (-0.051, 0.210) | **-1.923 (-3.212, -0.634)**** | -0.021 (-0.161, 0.120) |
|  | CMI | -0.002 (-0.003, -0.001)** | 0.002 (-0.011, 0.014) | 0.003 (0.002, 0.005)*** |
|  | PPC | -0.029 (-0.377, 0.320) | 1.782 (-2.507, 6.072) | -0.351 (-0.738, 0.037) |
|  | PC | 1.382 (-0.517, 3.281) | 18.195 (-0.287, 36.677) | -0.818 (-2.746, 1.110) |
|  | NPC | 0.505 (0.022, 0.987)* | 2.131 (-2.399, 6.662) | -0.003 (-0.488, 0.483) |

Note: *$p<0.05$, **$p<0.01$, ***$p<0.001$. The outcome variables highlighted in bold exhibited significant changes during the month of DIP reform, with a change range surpassing the actual change range observed in October 2021.

**Table S7** Robustness checks: the proportion of cases with point volumes below 500 and exceeding 2000 as two alternative outcome variables for CMI

| Hospital-Level | Variables | Baseline slope β_1_ (95%CI) | Step change β_2_ (95%CI) | Slope change β_3_ (95%CI) |
| --- | --- | --- | --- | --- |
| Tertiary | P500 | 0.392 (0.277, 0.507)*** | -3.520 (-5.878, -1.163)** | -0.746 (-0.958, -0.533)*** |
|  | P2000 | -0.534 (-0.636, -0.433)*** | 3.716 (0.877, 6.555)* | 0.982 (0.723, 1.240)*** |
| Secondary | P500 | 0.370 (0.180, 0.559)*** | -4.029 (-7.842, -0.216)* | -1.065 (-1.440, -0.690)*** |
|  | P2000 | -0.046 (-0.079, -0.013)** | 0.428 (-0.470, 1.326) | 0.168 (0.076, 0.261)** |
| Primary | P500 | 0.152 (-0.073, 0.376) | 0.745 (-3.307, 4.798) | -1.025 (-1.399, -0.651)*** |
|  | P2000 | -0.032 (-0.062, -0.003)* | 0.617 (0.230, 1.003)** | 0.044 (0.013, 0.075)** |

Note: *$p<0.05$, **$p<0.01$, ***$p<0.001$.

**Table S8** Robustness checks: removed data from cancer hospitals and psychiatric hospitals

| Hospital-Level | Variables | Baseline slope β_1_ (95%CI) | Step change β_2_ (95%CI) | Slope change β_3_ (95%CI) |
| --- | --- | --- | --- | --- |
| Tertiary | PRCP | -0.425 (-0.725, -0.124)** | 2.894 (-1.214, 7.002) | 1.287 (0.800, 1.774)*** |
|  | CMI | -0.010 (-0.014, -0.006)*** | 0.095 (0.023, 0.167)* | 0.021 (0.011, 0.031)*** |
|  | PPC | 0.160 (0.100, 0.219)*** | -0.637 (-1.399, 0.124) | -0.314 (-0.381, -0.247)*** |
|  | PC | 0.463 (0.255, 0.670) | 0.276 (-3.020, 3.573) | -1.297 (-1.629, -0.964)*** |
|  | NPC | 0.335 (0.083, 0.587)* | -1.961 (-4.601, 0.680) | -0.813 (-1.131, -0.495)*** |
| Secondary | PRCP | -0.170 (-0.351, 0.010) | -0.522 (-3.753, 2.709) | 0.600 (0.343, 0.857)*** |
|  | CMI | -0.005 (-0.005, -0.004)*** | 0.068 (0.034, 0.102)*** | 0.011 (0.007, 0.016)*** |
|  | PPC | -0.075 (-0.210, 0.060) | 5.797 (1.030, 10.564)* | -1.061 (-1.503, -0.620)*** |
|  | PC | -1.111 (-1.435, -0.788)*** | 14.850 (9.817, 19.883)*** | 0.591 (0.130, 1.053)* |
|  | NPC | 0.044 (-0.159, 0.247) | -4.277 (-7.964, -0.589)* | 0.762 (0.412, 1.112)*** |
| Primary | PRCP | -0.239 (-0.348, -0.129)*** | -7.074 (-11.057, -3.037)** | 0.916 (0.532, 1.300)*** |
|  | CMI | -0.013 (-0.025, -0.001)* | 0.029 (0.017, 0.040)*** | 0.003 (0.001, 0.004)** |
|  | PPC | 0.034 (-0.053, 0.121) | 1.477 (-1.500, 4.453) | -0.616 (-0.905, -0.327)*** |
|  | PC | 1.942 (1.417, 2.466)*** | 6.756 (1.080, 12.432)* | -1.765 (-2.359, -1.171)*** |
|  | NPC | 0.650 (0.501, 0.799)*** | 0.087 (-3.101, 3.275) | 0.040 (-0.276, 0.356) |

Note: *$p<0.05$, **$p<0.01$, ***$p<0.001$.
