# Supplementary material for: Does a new case-based payment system promote the construction of the ordered health delivery system? Evidence from a pilot city in China
Source: Int J Equity Health. 2024 Mar 14;23:55. doi: 10.1186/s12939-024-02146-y (PMC10938765; doi:10.1186/s12939-024-02146-y)
Supplement: Supplementary file 3 — Supplementary Materia 3. [file 12939_2024_2146_MOESM3_ESM.docx]

**Fig.S1** The impact of DIP reform on the total point volume of medical institutions in Tai’an using ITSA


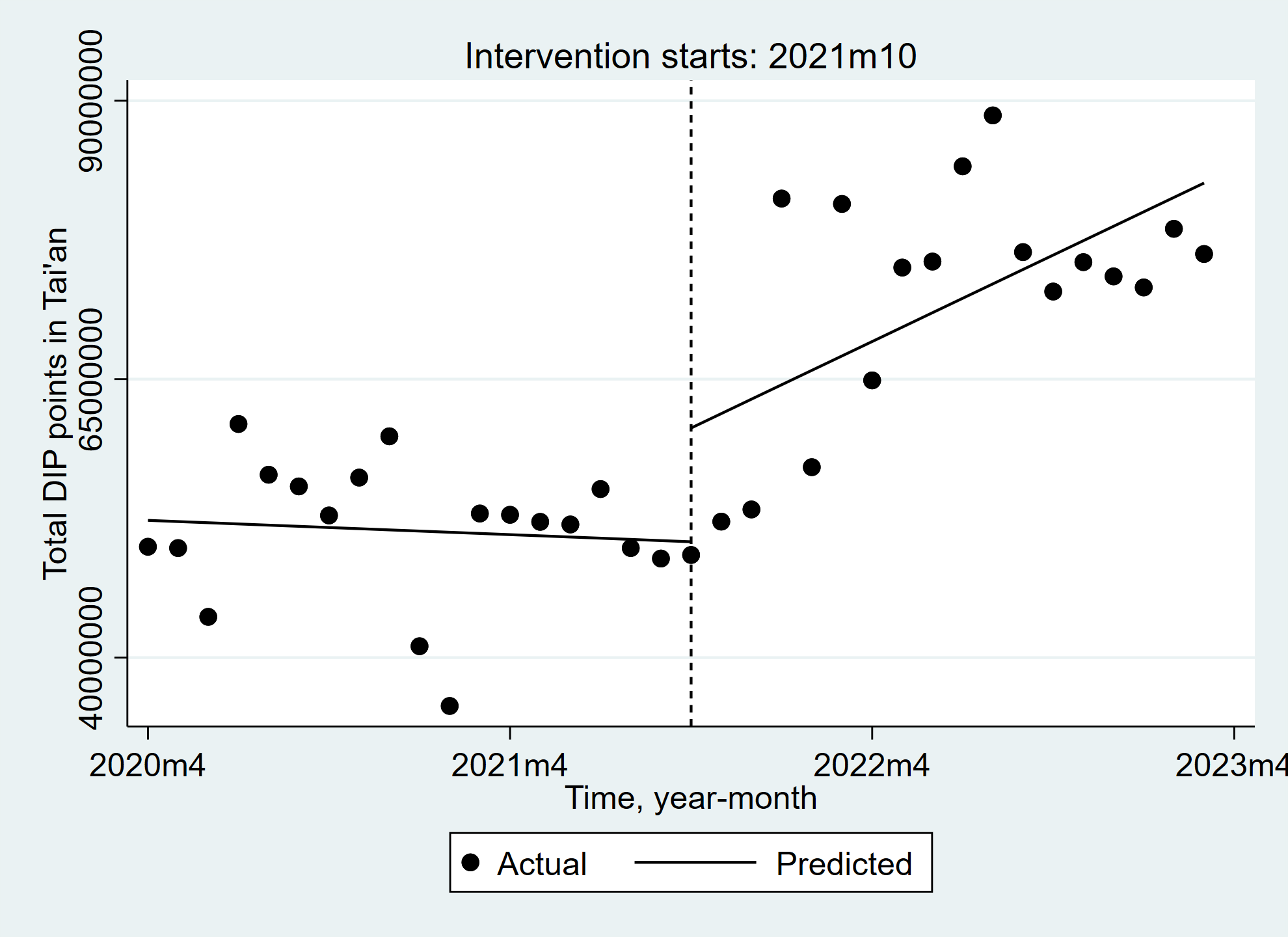


Note: Baseline slope β_1_=-106694 (P=0.602); Step change β_2_=10192959 (P=0.076); Slope change β_3_=1400489 (P=0.006).
