# Supplementary material for: Does a new case-based payment system promote the construction of the ordered health delivery system? Evidence from a pilot city in China
Source: Int J Equity Health. 2024 Mar 14;23:55. doi: 10.1186/s12939-024-02146-y (PMC10938765; doi:10.1186/s12939-024-02146-y)
Supplement: Supplementary file 4 — Supplementary Material 4. [file 12939_2024_2146_MOESM4_ESM.docx]

**Table S9** Results of the Newey-West standard errors and the Durbin-Watson statistic based on interrupted time series analysis

| Hospital-Level | Variables | N-W | | | D-W |
| --- | --- | --- | --- | --- | --- |
|  |  | β_1_ | β_2_ | β_3_ |  |
| Tertiary | PRCP | 0.028 | 0.445 | 0.051 | 1.838 |
|  | CMI | 0.002 | 0.028 | 0.003 | 1.445 |
|  | PPC | 0.042 | 0.545 | 0.050 | 1.462 |
|  | PC | 0.174 | 2.170 | 0.206 | 1.688 |
|  | NPC | 0.108 | 1.447 | 0.179 | 1.977 |
| Secondary | PRCP | 0.041 | 0.572 | 0.054 | 1.417 |
|  | CMI | 0.001 | 0.011 | 0.001 | 2.366 |
|  | PPC | 0.088 | 1.599 | 0.158 | 1.478 |
|  | PC | 0.203 | 2.534 | 0.288 | 1.634 |
|  | NPC | 0.109 | 1.613 | 0.144 | 2.090 |
| Primary | PRCP | 0.038 | 0.521 | 0.053 | 1.001 |
|  | CMI | 0.001 | 0.005 | 0.001 | 1.785 |
|  | PPC | 0.058 | 1.788 | 0.171 | 1.126 |
|  | PC | 0.367 | 4.372 | 0.432 | 0.794 |
|  | NPC | 0.074 | 0.863 | 0.085 | 0.700 |

Note: N-W and D-W denote the Newey-West standard errors and the Durbin-Watson statistic, respectively.
